# Supplementary material for: A UMLS-based spell checker for natural language processing in vaccine safety
Source: BMC Med Inform Decis Mak. 2007 Feb 12;7:3. doi: 10.1186/1472-6947-7-3 (PMC1805499; doi:10.1186/1472-6947-7-3)
Supplement: Additional file 13 — RAP application directory. Sets up directory for RAP (RDF application for PHP) source code [file 1472-6947-7-3-S13.gz › rap/doc/phpdoc/model/BlankNode.html]

xml version="1.0" encoding="iso-8859-1"?


Docs For Class BlankNode


## Class BlankNode

Description

Description |
Methods (details)

An RDF blank node.

In model theory, blank nodes are considered to be drawn from some set of 'anonymous' entities which have no label but are unique to the graph. For serialization they are labeled with a URI or a \_:X identifier.

History:

- 07-27-2003 : BlankNode can now receive both MemModel and DbModel as parameter
- 02-21-2003 : getLabel() added.
- 09-10-2002 : First version of this class.

- author: Chris Bizer <chris@bizer.de>
- access: public
- todo: nothing
- version: V0.9.1

Located in /model/Blanknode.php (line 29)

```
Object
   |
   --Node
      |
      --Resource
         |
         --BlankNode
```

Method Summary

Description |
Methods (details)

BlankNode
BlankNode
(mixed $namespace\_or\_uri\_or\_model, [string $localName = NULL])

boolean
equals
(object resource $that)

string
getID
()

string
getLabel
()

string
toString
()

Methods

Description |
Methods (details)

Constructor BlankNode (line 40)

Constructor You can supply a label or You supply a model and a unique ID is gernerated

- todo: nothing
- access: public

BlankNode

BlankNode
(mixed $namespace\_or\_uri\_or\_model, [string $localName = NULL])

- mixed
  $namespace\_or\_uri\_or\_model
- string
  $localName

equals (line 95)

Checks if two blank nodes are equal.

Two blank nodes are equal, if they have the same temporary ID

- access: public

boolean

equals
(object resource $that)

- object resource
  $that

---

Redefinition of:

Resource::equals()
:   Checks if the resource equals another resource.

getID (line 63)

Returns the ID of the blank node.

- access: public

string

getID
()

getLabel (line 72)

Returns the ID of the blank node.

- access: public

string

getLabel
()

---

Redefinition of:

Resource::getLabel()
:   Returns the label of the resource, which is the URI of the resource.

toString (line 82)

Dumps bNode.

- access: public

string

toString
()

---

Redefinition of:

Resource::toString()
:   Dumps resource.

#### Inherited Methods


Inherited From Resource

> Resource::Resource()  
> Resource::equals()  
> Resource::getLabel()  
> Resource::getLocalName()  
> Resource::getNamespace()  
> Resource::getURI()  
> Resource::toString()

Inherited From Object

> Object::toString()

Documentation generated on Thu, 7 Jul 2005 13:41:09 +0200 by phpDocumentor 1.3.0RC3
